# Comparative effectiveness of two popular weight loss programs in women I: body composition and resting energy expenditure

**DOI:** 10.1186/1550-2783-8-S1-P4

**Published:** 2011-11-07

**Authors:** Michelle Mardock, Brittanie Lockard, Jonathan Oliver, Mike Byrd, Sunday Simbo, Andrew Jagim, Julie Kresta, Claire Baetge, Peter Jung, Majid Koozehchian, Deepesch Khanna, Mike Greenwood, Chris Rasmussen, Richard Kreider

**Affiliations:** 1 Exercise & Sport Nutrition Lab. Texas A&M University, College Station, TX 77843, USA

## Background

A number of commercial diet and exercise programs are promoted to help people lose weight and improve fitness. However, few studies have compared the effects of following different types of exercise and diet interventions on weight loss. The purpose of this study was to compare the efficacy of a more structured meal plan based diet intervention and supervised exercise program to a traditional point based diet program with weekly counseling and encouragement to exercise.

## Methods

Fifty-one sedentary women (35±8 yrs, 163±7 cm; 90±14 kg; 47±7% body fat, 34±5 kg/m^2^) were randomized to participate in the Curves (C) or Weight Watchers (W) weight loss programs for 16-wks. Participants in the C program were instructed to follow a 1,200 kcal/d diet for 1-week, 1,500 kcal/d diet for 3 weeks, and 2,000 kcals/d diet for 2-weeks consisting of 30% carbohydrate, 45% protein, and 30% fat. Subjects then repeated this diet. Subjects also participated in the Curves circuit style resistance training program 3 days/week and were encouraged to walk at brisk pace for 30-min on non-training days. This program involved performing 30-60 seconds of bi-directional hydraulic-based resistance-exercise on 13 machines interspersed with 30-60 seconds of low-impact callisthenic or Zumba dance exercise. Participants in the W group followed the W point-based diet program, received weekly counseling at a local W facility, and were encouraged to increase physical activity. Dietary records, the International Physical Activity Questionnaire (IPAQ), dual energy X-ray absorptiometer (DEXA) determined body composition, and fasted resting energy expenditure (REE) measurements were obtained at 0, 4, 10, & 16 weeks and analyzed by multivariate analysis of variance (MANOVA) with repeated measures. Data are presented as changes from baseline for the C and W groups, respectively, after 4, 10, and 16 weeks.

## Results

Participants in the W group reported a greater reduction energy intake (C -270±450, -364±443, -386±480; W -636±510, -610±524, -549±522 kcals/d, p*_q_*=0.008) from baseline levels (C 1,693±430; W 1,954±524 kcals/d) with carbohydrate intake higher (19.6±11 grams/d, 6.0±1.9 %) and protein intake lower (-14.4±4 grams/d, -4.2±1 %) in the W group. Changes in group mean IPAQ walking (241±366 MET-min/wk, p=0.50), moderate PA (177±347MET-min/wk, p=0.61), vigorous PA (502±122 MET-min/wk, p=0.001), and total PA (925±587MET-min/wk, p=0.12) were higher in the C group. A significant overall MANOVA time (p=0.001) and diet (p=0.01) effect was seen in body composition results. Univariate analysis revealed that both groups lost a similar amount of weight (C -2.4±2.1, -4.4±3.6, -4.9±4.0; W -2.7±1.3, -5.3±2.4, -6.2±4.1 kg, p=0.31). However, fat mass loss (C -3.9±5.5, -4.6±5.3, -6.4±5.9; W -0.4±5.7, -2.1±6.7, -2.9±7.8 kg, p=0.09) and reductions in percent body fat (C -3.3±5.2, -3.2±4.6, -4.7±5.4; W 0.6±6.7, -0.6±8.3, -1.4±8.1 %, p*_q_*=0.054) tended to be greater in the C group while fat free mass was increased in the C while decreasing in the W group (C 1.5±4.3, 0.5±3.7, 1.3±4.0; W -1.8±5.4, -2.4±5.8, -2.5±5.1 kg, p=0.01). REE values increased over time in both groups and were non-significantly higher in the C group (C 0.9±2.2, 1.4±2.3, 1.3±1.9; W 0.6±2.0, 0.7±2.0, 0.6±2.3 kcals/kg/d, p=0.19).

## Conclusion

Results indicate that 16-wks of participation in the C program that involved a more structured meal plan based diet and supervised exercise program promoted more favorable changes in body composition than participation in the W program that involved adherence to a point based diet, weekly counseling, and encouragement to increase physical activity.

## Funding

Supported by Curves International (Waco, TX)

